# Enhancing workplace digital learning by use of the science of learning

**DOI:** 10.1371/journal.pone.0206250

**Published:** 2018-10-24

**Authors:** Kana Okano, Jakub R. Kaczmarzyk, John D. E. Gabrieli

**Affiliations:** 1 MIT Integrated Learning Initiative, Massachusetts Institute of Technology, Cambridge, MA, United States of America; 2 Department of Brain and Cognitive Sciences, Massachusetts Institute of Technology, Cambridge, MA, United States of America; 3 McGovern Institute for Brain Research, Massachusetts Institute of Technology, Cambridge, MA, United States of America; Waseda University, JAPAN

## Abstract

Digital learning is becoming the most commonly used portal for workplace learning, but its effectiveness is not clearly understood. We studied 99 employees on-site in a large company as they watched an already used and required training video. Employees were randomly assigned to one of four conditions: (1) a baseline condition of watching the video as in current practice; (2) a spontaneous discussion condition in which participants discussed the video with colleagues immediately after the video without any guidelines; (3) a structured discussion condition in which participants discussed the video with colleagues immediately after the video with an instructor guiding discussion topics; and (4) a testing condition in which test questions were interpolated throughout the video. Memory for the content of the video was measured on a recognition memory test completed 20–35 hours after watching the video. Employees who were in the interpolated-testing or structured discussion conditions had significantly superior memory for the video content (26% and 25% better respectively) relative to typical video viewing; spontaneous discussion did not enhance memory for content. These findings demonstrate that interpolated testing and structured discussion enhance information retention in the workplace and point to how learning science may accelerate workplace learning more generally.

## Introduction

Organizations invest substantial resources in learning and development to enhance their human capital. In 2015, US based companies spent $1,252 on average per employee for direct learning and development expenditures, making it a 180 billion dollar annual business in the US alone and growing every year [1]. The annual increases in investment in learning and development is also matched by annual increases in employees’ average hours spent on training.

Instructor-led live classroom delivery continues to be the most popular method of formal workplace learning, occupying 49% of all learning content [1]. However, with globalization and technological advances, companies are shifting to a more cost-effective content delivery method. In 2015, 41% of all learning content was delivered using technology—quickly approaching the amount of traditional classroom delivery.

Despite increasing investment of time and resources on creating and curating digital learning content, it is unclear how much of the learning done in this space is retained and carried over to impact work. In a corporate environment where fiscal success (return on investment) is perhaps the most important metric, there is surprisingly little scientific evidence about how well digital learning enhances performance. An initial step in such a scientific analysis is measuring how well the content of digital learning is remembered, because what is forgotten cannot be applied to work performance. Furthermore, such a scientific analysis could include measurement of whether specific methods could be applied to enhance long-term memory for the content of the digital courseware.

Our goal was to scientifically investigate how to improve the effectiveness of workplace digital learning by measuring the impact of two science-of-learning techniques thought to enhance information retention that can be easily applied in the workplace learning environment. Collaborating with a large corporation, we examined information retention in employees for a regularly used training video on development and operation. Employees were randomly assigned to one of four training conditions –1. a baseline conditioning with watching the video as usual, 2. Spontaneous peer discussion, 3. Structured peer discussion, or 4. Interpolated testing. The latter three conditions driven from the science-of-learning and are described in detail below. Then, 20–30 hours later, returning employees were administered a test to measure how much of the video content was remembered and retained in long-term memory. The critical question was whether any of the science-of-learning manipulations would enhance information retention relative to typical practices.

### Peer discussion–Spontaneous or structured

One science-of-learning technique that can improve learning outcome is peer discussion. Research in higher education suggests that peer discussion fosters deep and lasting learning outcome [2]. This is thought to emerge through active engagement with the content being learned, which allows learners to discuss and construct their own understanding of the material [3–6].

Peer discussion is traditionally carried out as spontaneous discussion where students discuss relevant topics without any formal structure or guidance. One such technique was introduced by Mazur [6] where students in a traditional classroom were asked content questions regarding the lecture. After answering these questions, but before given the correct answer, students were prompted to discuss how they came to their answers with peers sitting nearby. When the same question was asked again after the spontaneous discussion, accurate memory for the content increased. These results have been replicated in multiple studies [4,7], showing the effectiveness of spontaneous peer discussion.

Extending this research, some have found that peer discussion can further enhance learning if accompanied by instructors’ cues (structured discussion) [8,9], rather than having students engage in spontaneous discussions. Although most of these studies involve problem-solving tasks in addition to peer discussions, the peer discussions themselves may encourage active engagement that enhances learning.

The idea that discussion is beneficial in learning is represented in flipped classrooms, in which students view video lectures independently and asynchronously and then engage in collaborative learning through active problem solving or discussions during traditional classroom time. The very concept of flipped learning stems from the idea that classroom time is better spent actively discussing the content, rather than passively listening to a lecture.

Research in flipped learning has shown that students’ perception of learning effectiveness is highly positive, and more importantly, students show better performance as evaluated with examinations. This has been observed in all levels of education from high school [10,11], to higher education [12,13], to professional education[14,15].

### Interpolated testing

Historically, quizzes and tests have been frequently used to assess learning, but rarely used to enhance learning itself. Numerous studies have shown that any attempt to retrieve information by testing will increase the likelihood of remembering that information in the long term [16–19]. This phenomenon is known as the “testing effect.” The facilitative effect of testing on learning is thought to be a result of the act of retrieval strengthening the link between cues and targets [20]. This link is unique to testing and cannot be gained through other forms of study as research has shown that memory gained through testing outlasts those gained through repeated study[18].

Interpolated testing is a learning technique that quizzes the learner intermittently during the learning process. Aside from the benefits of greater long-term retention, interpolated testing can also reduce the learner’s instances of mind wandering away from the material and help the learner stay on task [21,22]. Although interpolated testing has been studied in laboratories using lists of words [23,24] and online university lectures [22,25], it has never been studied in a workplace setting.

Our aim was to examine science-of-learning techniques that can be easily supplemented to existing workplace digital courses and compare their effectiveness relative to the current practice of simply watching the video. We hypothesized that both interpolated testing and peer discussion would enhance information retention compared to baseline. Based on previous research, we also hypothesized that peer discussion would be more effective when it is structured rather than spontaneous.

## Materials and methods

### Participants

One hundred fifty-three randomly selected employee volunteers (81 females) from Accenture PLC (Accenture) participated in the two-day experiment. Employees were informed of the study and gave written consent obtained in accordance with guidelines of the 1964 Declaration of Helsinki and approved by Massachusetts Institute of Technology (MIT) Committee on the Use of Humans as Experimental Subjects. Participants in this study were either part of a 1~2 week corporate training session which took place in St. Charles, Illinois, or taking part in an Accenture organized short program offered at MIT. Accenture is a leading global professional services company with over 401,000 people serving clients in more than 120 countries and operates in over 200 cities across 120 countries.

Fifty-four participants were excluded from analysis because they did not complete the second day of assessment. Due to company policy, we were only able to ask which age group they belonged in, rather than their actual age. The demographics of the 99 participants (45 females) who completed the two-day experiment are detailed in Table 1. The large percentage of attrition (35%) was likely due in part to participants being actual employees in a workplace environment who were busy with workplace tasks and unable to return for the second day of assessments.

**Table 1:** Participants in the experiment.

Age Range	Number of Females	Number of Males	Total
21–25	19	15	34
26–30	19	26	45
31–35	3	10	13
36–40	1	1	2
41–45	2	2	4
46–50	1	0	1
56–60	0	1	1

Age range and gender of all participants who completed the two day experiment.

### Procedure

On Day 1 of the study, participants were asked to watch and learn an eight-minute 20-second introductory lecture on development and operations (DevOps), a practice that emphasizes the effective delivery of information-technology (IT) products through active collaboration of software developers and other IT professionals (see transcript of entire video made available at https://github.com/kokano/Workplace-Digital-Learning/Video_Transcript.docx) This video has been used regularly for training purposes within the Accenture organization. Volunteers were randomly assigned to one of four conditions: Video, Spontaneous Discussion, Structured Discussion, or Interpolated Testing. Although viewing of the video was a requirement of the workplace, returning for testing of what had been retained in memory was optional. Of the 54 participants who did not return for Day 2 of the task, 22 were in the Video Group, 13 in the Spontaneous Discussion Group, 6 in the Structured Discussion Group, and 13 in the Interpolated Testing Group. The number of people who dropped out of the study did not differ by condition, *X*^2^ (3, N = 154) = 4.30, *p* = 0.23.

The Video Group (n = 28, Female = 11) simply watched the video in the typical manner to measure the effectiveness of current practice. The Spontaneous Discussion Group (n = 20, Female = 9) was informed ahead of time that they would be discussing the content with two to four other colleagues immediately after watching the video. No further instructions as to what should be discussed were stipulated and the discussion content was not explicitly monitored. However, participants largely stayed on topic and discussions lasted no longer than 10 minutes. Those who veered off topic were discussing matters related to but not directly pertaining to the video content. Everyone in the group participated in these discussions. The Structured Discussion Group (n = 19, Female = 11) engaged in a structured discussion with an experimenter who followed topics that were pertinent to the main points made in the video. The only cues provided by the experimenter were: 1. What do you think were the key points of the video? 2. What do you think are the benefits of DevOps? 3. How do those help make DevOps effective? (Follow up question to 2.) Everyone participated in these discussions and lasted no longer than 10 minutes. The Interpolated Testing Group (n = 33, Female = 16) had multiple-choice questions interspersed throughout the video approximately every minute asking content questions regarding the part of the video they had just viewed. These questions were self-timed (i.e., without a time limit). Once participants selected their answer, a green check mark appeared next to the correct answer giving them immediate feedback. The screen that displayed the correct answer stayed on the screen until the learner pressed a button to move on to the next section of the video. Almost all of the participants were novices in this topic and only two reported as having some exposure to the topic in the past. These two participants were in the Video Group and Interpolated Testing Group respectively.

Next day, approximately 20–35 hours later, (Day 2), participants completed an online post-test that tested their memory (knowledge) on the content of the video. The reason we waited a day to conduct the post-test stems from the research on the Ebbinghaus forgetting curve [26–28]. The original research as well as repeated replications have indicated that memory declines rapidly in the first 9 hours and quickly plateauing thereafter. Therefore, recall test 1 day later is a reliable measure of what people will remember up to a month in the future. Because most information acquired from work related videos are not used immediately after viewing, we elected to test people’s acquisition of video information one day later.

**Fig 1:**
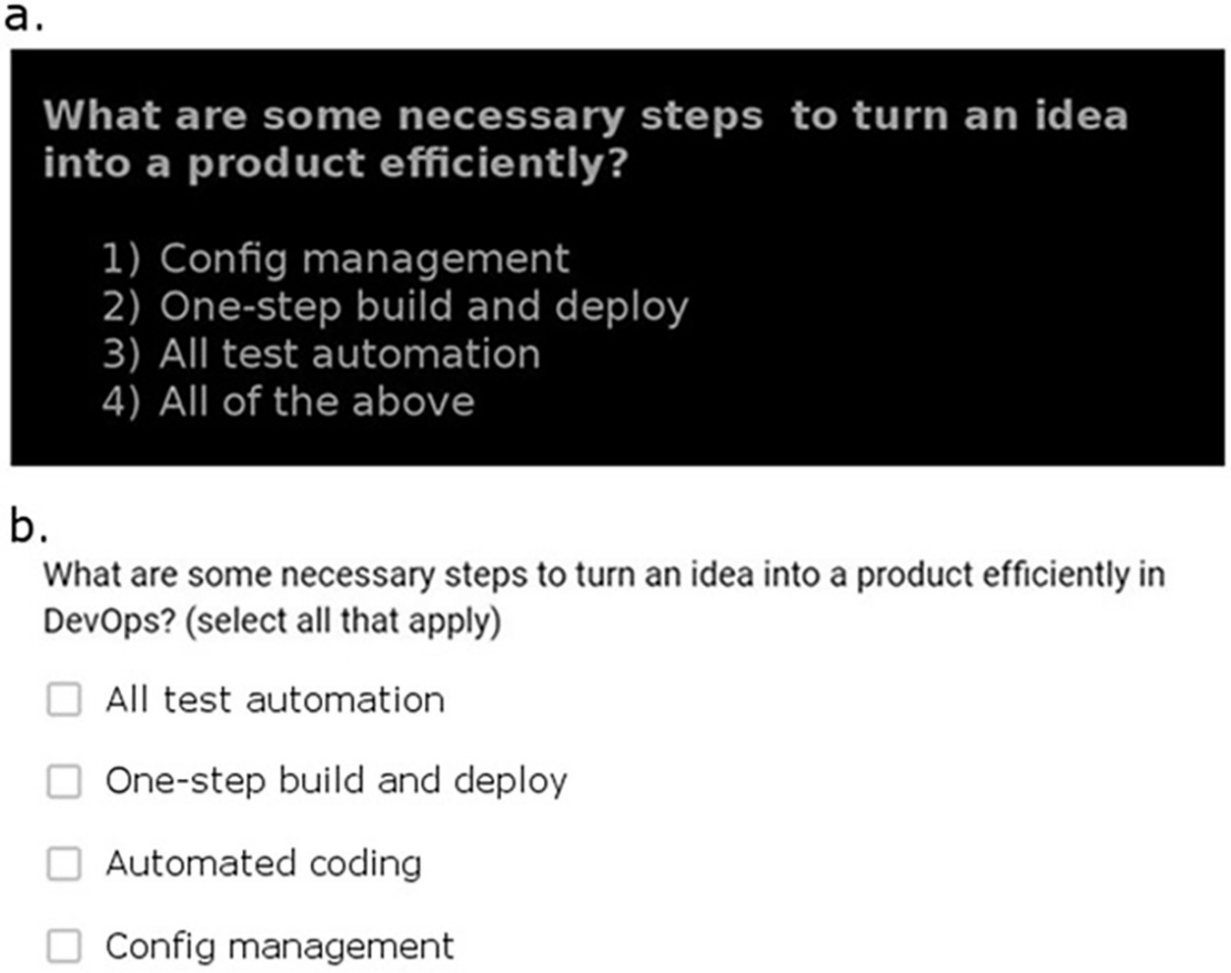
Example questions. **a:** Example interpolated question during the video. **b:** Example post-test question for the same question representing a different format of questioning.

The post-test included three free-form questions and 16 multiple-choice questions. Six of the 16 multiple-choice questions were presented to the Interpolated Testing Group on the first day, but three of these six questions were asked in a different format than in the interpolated testing (see Fig 1 for example). The remaining 10 multiple-choice questions and all three free-form questions were novel to all participants. There was no time limit on the post-test.

### Scoring

The 10 multiple-choice items that had only one correct answer were awarded one point per item. For the six multiple choice questions that asked learners to “select all that apply”, one point was awarded to the item only if learners had selected all of the correct answers. No points were awarded if they had lacked any choices or if they had selected any incorrect choices. For short-answer questions, a response was coded as correct and given one point if it included key phrases.

## Results

### Post-test

**Fig 2:**
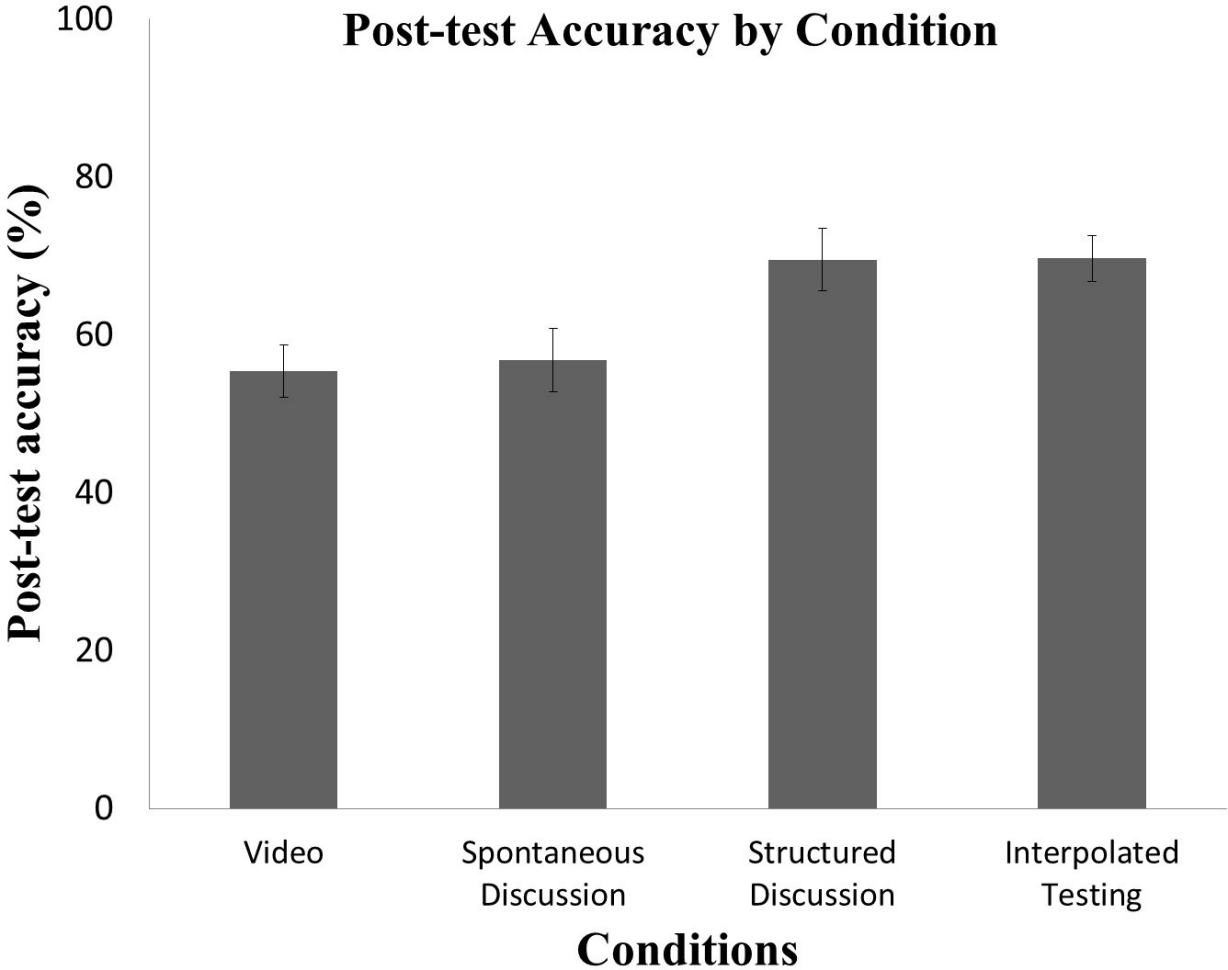
Post-test accuracy by condition. Average post-test performance (percentage correct) as a function of condition. Error bars represent standard errors of the mean.

In the post-test, the Video Group had a mean score of 55.45% (SD = 17.57%), the Spontaneous Discussion Group had a mean score of 56.84% (SD = 17.96%), the Structured Discussion Group had a mean score of 69.53% (SD = 17.15%), and the Interpolated Testing Group had a mean score of 69.70% (SD = 16.85) (Fig 2). For participants in the Interpolated Testing Group, accuracy during interpolated testing significantly correlated and positively with post-test scores on the same items (r (33) = 0.36, *p* = 0.04), and correlations with items that were not tested also trended towards significance (r (33) = 0.30, *p* = 0.09).

We conducted an ANOVA using post-test scores as the dependent variable, Condition (Video/Spontaneous Discussion/Structured Discussion/Interpolated Testing) as the independent variable. The ANOVA revealed a significant main effect of Condition (F (3, 96) = 5.15, *p* = 0.002), such that the Interpolated Testing Group outperformed the Video (*p* = 0.01) and Spontaneous Discussion (p = 0.049) Groups as revealed by a Tukey HSD. The Structured Discussion Group also outperformed the Video Group (p = 0.036) but not the Spontaneous Discussion Groups (p = 0.108). Neither the Video and Spontaneous Discussion Groups nor the Structured Discussion and Interpolated Testing Groups differed reliably from one another (all *ps* > 0.99). Restricting the analyses to questions that differed from the interpolated test questions resulted in similar outcomes revealing a significant main effect of Condition (F (3,96) = 4.15, *p* = 0.008).

## Conclusions

We report the first empirical evidence that interpolated testing and structured discussions enhance long-term retention of the content of video training in the workplace. Testing for knowledge retained 20–35 hours after study revealed that participants who participated in structured discussion after the video or who answered questions interspersed throughout the video remembered the content substantially more (25~26% respectively) than participants who simply watched the video as in current practice. Unstructured spontaneous discussion did not, however, enhance information retention significantly above baseline training as usual.

The effect of interpolated testing on long-term retention of information from the training video is consistent with the experimental literature that intermittent testing is a powerful way to enhance on-line learning [21,25,29]. Indeed, digital learning provides an exceptional platform for interpolated testing because such testing can be carefully and consistently interpolated between content. The present study shows for the first time that interpolated testing enhances information retention not only in laboratory studies of arbitrary materials but also in the workplace with mandated content.

A novel finding from this study was that structured discussion enhanced digital learning but that spontaneous discussion did not. The structured discussion followed key points of the video and gave cues to the learners on what was important to be learned, as well as a chance to retrieve relevant content immediately after watching the video. Thus, structured discussion offered a second, explicit, and reinforcing learning experience on the content. Enhanced learning following structured discussion is consistent with previous literature that found peer discussion to be more effective when accompanied by instructors’ cues [8,9].

Although both interpolated testing and structured discussion significantly and similarly enhanced digital learning, interpolated testing has the advantage from the viewpoint of practical application. It costs less and is easier to implement with high fidelity than structured discussion, which costs more for paying an instructor, takes more time for employees, and may require a skilled instructor. Although it may have been hypothesized that the human interaction in structured discussion would engage social or cognitive mechanisms beyond those engaged by interpolated testing, in fact the automated interpolated testing was equally effective in enhancing learning beyond current workplace practice. This finding contrasts with other examples in which contingent human interaction enhances learning better than non-contingent teaching, such as viewing a video for children learning Mandarin Chinese [30].

A major strength of this study was that it occurred in an actual workplace and participants were employees viewing a video that was already a workplace requirement (i.e., it had ecological validity). This strength, however, came with two major limitations. First, given the actual workplace use of the video, we could not include comparison conditions that would provide insights into the mechanisms of enhanced learning but were unlikely to enhance learning or could even depress learning below current workplace practice. For example, we could have included a condition with an unrelated interpolated task to demonstrate that the content of the interpolated tasks and had to be relevant to the video to enhance. This would, however, have been nonsensical and distracting to employees fulfilling a job requirement.

Second, we had a high rate of attrition in regards to employees who did not complete the test of retention on the day following the video training. This limitation reflected a trade-off between the strength of conducting the study in a real work environment with actual employees and actual digital learning and the drawback that employees were busy with tasks in the workplace. High attrition may have reflected the optional nature of completing the delayed test, and the lack of a specific motivation (e.g., participant payment) to complete the test. It is interesting to note, however, that the two highest performing groups had the lowest dropout rates. This suggests that the Structured Discussion and Interpolated Testing enhanced employee motivation if willingness to complete the study was an index of motivation. Enhanced motivation would be an added benefit of the two conditions that enhanced learning, and consistent with the idea that motivation and learning are strongly intertwined. Furthermore, if dropout reflected low motivation to learn the material, then our study may have underestimated the learning benefits because the Video and Spontaneous Discussion Groups may have self-selected such that individuals with low motivation were underrepresented in the those two worse learning groups.

In the present interpolated testing design, participants received feedback about whether each question was answered correctly or incorrectly, and for incorrect answers the correct answer was highlighted. Further, some of the final test questions overlapped with the interpolated test questions. Neither of these design aspects likely led to the learning gains from interpolated questions. First, many experiments have demonstrated that interpolated testing without corrective feedback enhances learning substantially more than other strategies for learning such as repeated study or other elaborative strategies [16,18,31]. Second, participants who received interpolated testing had about the same benefit for final test questions whether or not the final test questions matched the interpolated test questions. Indeed, the learning gains shown on the novel questions on the final test demonstrate that enhanced learning for all information, not just the intermittently tested information, occurred as a benefit of interpolated testing on only some of the information.

In summary, we identified two effective interventions (interpolated testing and structured discussion) that can be easily applied to workplace digital learning without much change to existing videos. This adds to the existing literature pointing to the effective science-of-learning techniques that can be applied to workplace learning.
